# Biliary Metabolome Profiling for Evaluation of Liver Metabolism and Biliary Tract Function Related to Organ Preservation Method and Degree of Ischemia in a Porcine Model

**DOI:** 10.3390/ijms24032127

**Published:** 2023-01-21

**Authors:** Kamil Łuczykowski, Natalia Warmuzińska, Dagmar Kollmann, Markus Selzner, Barbara Bojko

**Affiliations:** 1 Department of Pharmacodynamics and Molecular Pharmacology, Faculty of Pharmacy, Collegium Medicum in Bydgoszcz, Nicolaus Copernicus University in Toruń, 85-089 Bydgoszcz, Poland; 2 Department of Surgery, Ajmera Transplant Centre, Toronto General Hospital, University Health Network, Toronto, ON M5G 2C4, Canada; 3 Department of General Surgery, Medical University of Vienna, 1090 Vienna, Austria

**Keywords:** bile, liver transplantation, solid-phase microextraction (SPME), organ preservation, graft quality assessment, NEVLP, metabolomics

## Abstract

The development of surgical techniques, immunosuppressive strategies and new organ preservation methods have meant that transplant centers have to face the problem of an insufficient number of organs for transplantation concerning the constantly growing demand. Therefore, using organs from expanded criteria donors and developing new analytical solutions to find parameters or compounds that would allow a more efficient assessment of organ quality before transplantation are options for meeting this challenge. This study proposed bile metabolomic analysis to evaluate liver metabolism and biliary tract function depending on the organ preservation method and degree of warm ischemia time. The analyses were performed on solid-phase microextraction-prepared bile samples from porcine model donors with mild (heart beating donor [HBD]) and moderate warm ischemia (donation after circulatory death [DCD]) grafts subjected to static cold storage (SCS) or normothermic ex vivo liver perfusion (NEVLP) before transplantation. Bile produced in the SCS-preserved livers was characterized by increased levels of metabolites such as chenodeoxycholic acid, arachidonic acid and 5S-hydroxyeicosatetraeonic acid, as well as saturated and monounsaturated lysophosphatidylcholines (LPC). Such changes may be associated with differences in the bile acid synthesis pathways and organ inflammation. Moreover, it has been shown that NEVLP reduced the negative effect of ischemia on organ function. A linear relationship was observed between levels of lipids from the LPC group and the time of organ ischemia. This study identified metabolites worth considering as potential markers of changes occurring in preserved grafts.

## 1. Introduction

Transplantation is a very effective treatment for various forms of end-stage liver failure. Thanks to developments in surgical techniques, immunosuppressive strategies and patient-management approaches, the number of candidates eligible for liver transplantation is constantly growing; unfortunately, however, the number of donors remains constant and insufficient [1]. Transplantation using organs from expanded criteria donors (ECD) is one potential option for meeting this increasing demand. Although the use of ECD organs can significantly reduce wait times for transplantation, these organs also carry an increased risk of dysfunction or rejection [2,3]. Thus, the greatest challenge in contemporary transplantation is the development of an organ-preservation method that not only enables the organ to be maintained in optimal condition, but also allows its function to be assessed prior to surgery.

Due to its readily available logistics and low cost, static cold storage (SCS) is currently the standard technique for organ preservation in clinical practice. The low temperatures used during SCS delay metabolic processes, thus reducing ischemic injury [4,5]. However, ECD grafts appear to be more susceptible to prolonged ischemia, increased morbidity and mortality in post-liver-transplantation recipients [3]. Normothermic ex vivo liver perfusion (NEVLP) has emerged as an intriguing alternative to SCS, as it allows active metabolism to be maintained during the storage period. On the one hand, this approach results in increased energy consumption and oxygen demand, which makes the preservation procedure much more complex compared to cold preservation; on the other hand, it also minimizes the risk of liver damage and early allograft dysfunction (EAD) by reducing the exposure of grafts to static cold ischemia [6]. Since the liver exhibits metabolic activity during normothermic perfusion, one key advantage of this approach is that it enables the assessment of organ viability before transplantation. The literature contains numerous studies wherein liver function during perfusion has been assessed through the analysis of tissues, perfusate and bile [6,7,8].

In the present study, solid-phase microextraction (SPME) is used as a sample-preparation method to evaluate the metabolomic profile of bile from livers preserved via SCS and NEVLP. SPME has already been applied successfully in metabolomics studies, and its usefulness has been reported in many applications, including differentiating patients with bladder cancer [9], profiling brain tumors [10] and assessing the quality of organs intended for transplantation [11,12]. In addition, the effect of the organ’s ischemic time on the composition of bile was assessed to monitor changes in liver metabolism and biliary functions.

## 2. Results

**Figure 1:**
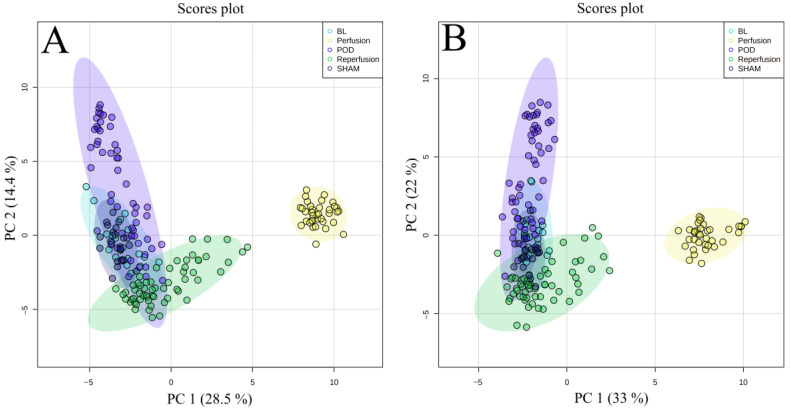
Score plots (PCA) showing separation indicating differences in the metabolomic patterns of the samples based on the time interval at which they were collected. (**A**) positive ionization mode, (**B**) negative ionization mode.

**Figure 2:**
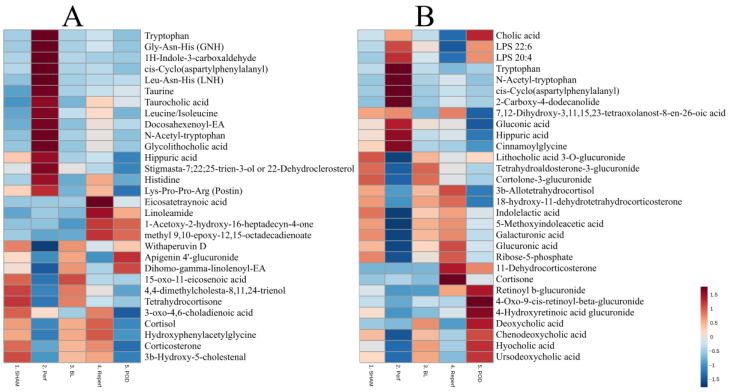
Heatmap showing the overall levels of the metabolites differentiating the samples collected at each time interval (Ward’s clustering algorithms, Euclidean distances). (**A**) Positive ionization mode, (**B**) negative ionization mode. The dark red squares on the heat map indicate a high abundance of that feature in a specific group of samples, whereas dark blue indicates a low abundance.

Principal component analysis (PCA) was performed to evaluate the instrument’s stability during the analyses. The compact clustering observed among the QC samples confirmed the quality of the obtained results (Figure S1A,B). Furthermore, the two-dimensional score plots (PC1 vs. PC2) showed an apparent separation indicating differences in the metabolomic patterns of the samples based on the time interval at which they were collected. The PCA plot for the bile samples revealed a clear separation between perfusion and the other samples along PC 1 and less separation between reperfusion and the POD sample groups along PC 2 (Figure 1A,B). ANOVA tests were used to identify the top 30 differentiating metabolites in the study group, with the overall changes in these compounds being visualized as heatmaps (Figure 2A,B). These metabolites primarily consisted of amino acids and lipids, including bile acids and steroid compounds, along with their derivatives. The results revealed that the perfusion samples—which, unlike the other samples, were collected under ex vivo conditions—possessed a different metabolic profile compared to the other samples.

A further, more detailed analysis of the results was subsequently conducted. This analysis was divided into three parts: (i) assessing changes in the metabolomic profile of bile after transplantation based on the method of organ preservation (i.e., SCS or NEVLP); (ii) determining how 30 min warm ischemia affected the metabolomic profile of bile after transplantation; and (iii) examining how the degree of warm ischemia influenced the bile metabolome in the peri-transplant period during the application of NEVLP.

### 2.1. Evaluation of the Metabolomic Profile of Bile in the Post-Transplant Period after Employing SCS and NEVLP

The Kruskal—Wallis test and multiple (post-hoc) mean-rank comparisons were employed to determine how SCS and NEVLP methods affected the bile metabolome. Analyses were performed for the HBD (heart beating donor) and 30′DCD (donation after circulatory death) groups in the samples collected after organ implantation. Samples collected during reperfusion (3 h and 5 h) and post-operative days 1 and 3 (POD1 and POD3, resepectively) were combined into larger groups to increase the readability and statistical significance of the obtained results. No significant differences in the composition of the bile samples collected immediately after transplantation (baseline) were observed among the studied groups. Rather, changes only appeared during ongoing reperfusion or a few days after surgery, affecting organs from both the HBD and 30′DCD donors. Samples that had been preserved via SCS were characterized by higher levels of lysophosphatidylcholines (LPC) and lysophosphatidylethanolamines (LPE). Additionally, the bile samples from the SCS group also contained higher levels of glycocholic acid (GCA), glycoursodeoxycholic acid (GUDCA), glycohyocholic acid (GHCA) and tauroursodeoxycholic acid (TUDCA) during reperfusion, and higher concentrations of chenodeoxycholic acid (CDCA) post-operation. In contrast, the organs from the NEVLP group showed significantly increased taurocholic acid (TCA) secretion into the bile during reperfusion. The differences in metabolite levels based on the preservation method used for the HBD and 30′DCD groups are summarized in Table 1 and Table 2 for positive and negative ionization mode, respectively. The largest number of differentiating compounds were identified during reperfusion, with 39 and 17 being isolated for the HBD and 30′DCD groups, respectively. By contrast, only 14 (HBD) and 6 (30′DCD) were identified post-operation. A high degree of change characterized not only bile acids, but also amino acids and their derivatives. Among the differentiating lipids, LPCs constituted the largest group.

### 2.2. Effect of 30 Min Ischemia on the Metabolomic Profile of Bile after Transplantation

**Figure 3:**
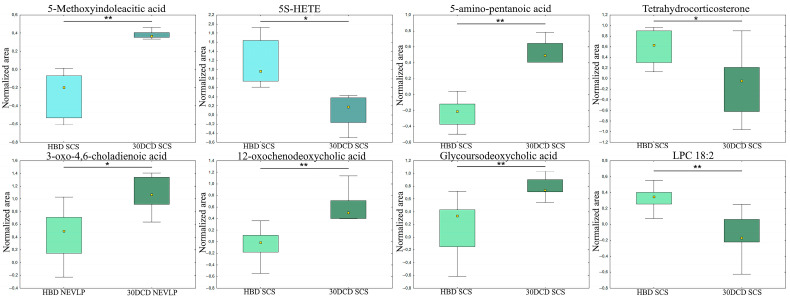
Selected metabolites differentiating the HBD and 30′DCD groups. The boxplots display the normalized peak areas, while the height of the rectangle represents the peak areas in the interquartile range (Q1 and Q3). The upper whisker denotes the largest data point excluding any outliers, and the lower whisker indicates the lowest data point excluding any outliers. The median normalized peak area of each group is indicated with a yellow square. *—*p* < 0.05; **—*p* < 0.01; blue = baseline; green = reperfusion.

Next, we examined how a 30 min warm ischemia affected the composition of bile produced by the transplanted organ. To this end, analyses were performed separately for the SCS and NEVLP groups, with the changes in the levels of the selected metabolites being shown in Figure 3. The obtained results showed that a 30 min ischemia had a greater impact on the function of the livers subjected to SCS. In this group, changes in metabolites became apparent immediately after transplantation. At the remaining time points, changes in the levels of numerous metabolites were observed in the SCS group, while differences were only observed in single compounds in the NEVLP group. In the SCS group, the bile secreted by the liver undergoing a short ischemia period was characterized by decreased levels of LPCs and increased levels of fatty amides and oxidized bile acids such as 3-oxo-4,6-choladienoic acid and 12-oxochenodeoxycholic acid. Moreover, the bile samples from the 30′DCD group exhibited lower levels of 5S-hydroxyeicosatetraeonic acid (5S-HETE) at baseline and tetrahydrocorticosterone during reperfusion, and elevated levels of 5-methoxyindoleacetic acid at baseline and 5-amino-pentanoic acid during reperfusion. A complete list of metabolites exhibiting differences due to undergoing ischemia is provided in Tables S1 and S2 for positive and negative ionization modes, respectively.

### 2.3. Influence of Degree of Ischemia on the Bile Metabolome in the Peri-Transplant Period during NEVLP

**Figure 4:**
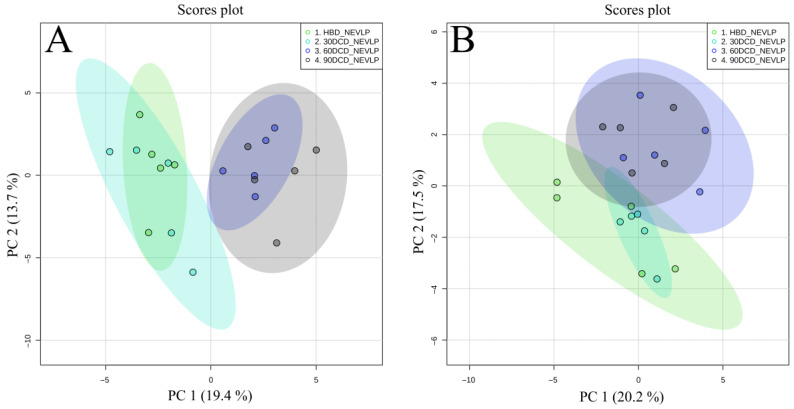
Score plots (PCA) showing the separation between groups with different ischemic times prior to organ harvest. (**A**) Positive ionization mode, (**B**) negative ionization mode.

**Figure 5:**
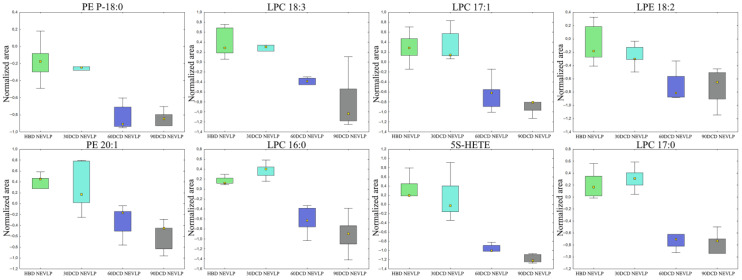
Selected compounds with levels linearly correlated with ischemia time prior to organ harvest. The boxplots show the normalized peak areas, and the height of the rectangles represent the peak areas in the interquartile range (Q1 and Q3). The upper whiskers indicate the largest data point, excluding any outliers, while the lower whiskers indicate the lowest data point, excluding any outliers. The median normalized peak area of each group is indicated with a yellow square.

Tests were conducted to assess how gradually increasing organ warm ischemia time affected the bile metabolome. To this end, chemometric analysis was performed to visualize the data and investigate differences in the metabolomic profiles of bile samples collected from livers in the HBD, 30′DCD, 60′DCD and 90′DCD groups prior to organ harvest (SHAM samples). The PCA results for the analyzed groups (Figure 4) show that prolonging the ischemia time affects the bile metabolome. Next, Spearman’s rank correlation was used to select compounds with levels that correlated linearly (R > 0.7) to organ ischemia time. The identified metabolites mainly consisted of LPCs, and their biliary levels were found to be negatively correlated with ischemia time (Figure 5). Detailed data relating to these correlations, including correlation coefficients and significance levels, are presented in Table S3.

Additionally, individual ischemic groups were compared with the HBD group using a Volcano plot, assuming differences with a fold change > 2 and *p* < 0.05 with false discovery rate (FDR) correction as statistically significant. The data capturing the number of metabolites differentiating particular groups at specific time intervals indicate that ischemia lasting up to 60 min does not significantly affect bile composition, and thus, only causes slight metabolic changes within the organ. However, more extensive changes in the bile metabolome were observed after 90 min of ischemia. These two groups (90′DCD vs. HBD) were differentiated by the highest number of metabolites during the perfusion period, with visible normalization at further time points. Table 3 shows the number of metabolites differentiating the moderate ischemic groups from the HBD group, while Figure S2 shows the identified metabolites distinguishing the 90′DCD group from the HBD group.

**Figure 6:**
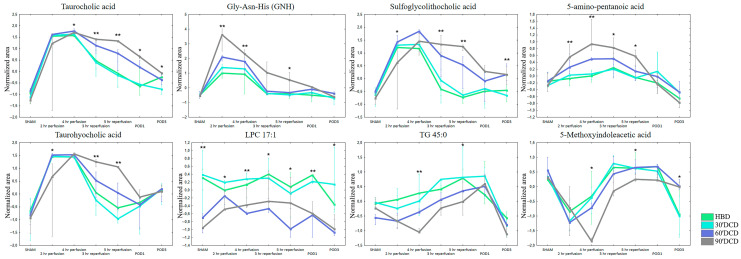
Changes in the levels of selected metabolites in the peri-transplant period. The plots display the medians of the normalized peak areas. The upper whisker denotes the largest data point, excluding any outliers, while the lower whisker indicates the lowest data point, excluding any outliers. *—*p* < 0.05; **—*p* < 0.01.

In addition to examining the effect of ischemia on metabolite concentrations in bile, levels of individual compounds were monitored throughout the peri-transplant period by taking samples at specific time points. Individual metabolites showed different concentration trends between organ harvest and transplantation. LPCs were consistently present at lower levels in bile produced by livers that had undergone prolonged ischemia. The most significant differences in the levels of the remaining metabolites, including bile acids (e.g., TCA, THCA, GLCA-sulfate), amino acids, peptides and others, were observed during perfusion and reperfusion with gradual equalization occurring post-operation. Selected compounds showing differences at individual phases of the peri-transplant period are illustrated in Figure 6.

## 3. Discussion

This study aimed to develop a new organ-preservation method that also allows for the assessment of graft quality prior to transplantation. To this end, metabolomic analysis of bile produced by livers in the peri-transplant period was conducted. Bile is not commonly used in scientific research, as it is difficult to prepare due to its consistency (density, thickness), high lipid content and high variability of physicochemical properties. However, the literature contains reports detailing the high scientific value of bile analysis, particularly as a matrix in proteomic and metabolomic analysis in patients with biliary tract diseases [13,14,15,16]. Furthermore, it has been proven that bile production during NEVLP is an important indicator of liver viability. Moreover, it is believed that, along with secretion levels, the composition of bile may also be of high importance [17]. Unfortunately, the literature on this topic predominantly focuses on the determination of total bile acids rather than individual bile acid profiles, and does not consider the other components of the bile metabolome. To the best of our knowledge, this is the first bile metabolome study to use SPME to compare different liver preservation methods and to evaluate how varying degrees of ischemia times impact bile composition.

The analysis of the metabolomic profiles of bile at particular time intervals revealed significant differences among the samples collected during perfusion (Figure 1). In particular, these samples contained higher concentrations of amino acids, including tryptophan, leucine, isoleucine and histidine, as well as some peptides (Figure 2). It is known that the administration of amino acids during transplantation has a nutritional and hepatoprotective effect on the transplanted organ [18]. Therefore, elevated levels of these metabolites in bile may be due to hepatic metabolism, or related only to the increased supply of amino acids in the perfusion fluid, as it is impossible to distinguish the origin of these compounds. In addition, the perfusion samples had a slightly different bile acid profile compared to the in vivo samples, especially in the post-transplant period, while the ex vivo samples showed significantly lower concentrations of individual unconjugated primary and secondary bile acids compared to the samples collected after transplantation. Primary bile acids are excreted into the bile mainly as taurine or glycine conjugates. After entering the intestine, they are deconjugated by the bile salt hydrolase (BSH) produced by bacteria, and some are converted to secondary bile acids and passively absorbed into the hepatic portal circulation [19]. Depending on their structure, they are then re-secreted into the bile unchanged or they undergo glucuronidation in the endoplasmic reticulum [20]. Therefore, the pool of these newly formed bile acids increases after organ implantation due to the restoration of enterohepatic circulation.

### 3.1. Evaluation of the Metabolomic Profile of Bile in the Post-Transplant Period after Employing SCS and NEVLP

In recent years, normothermic machine perfusion (NMP) has been increasingly used to preserve the liver before transplantation [21]. NMP’s advantages over routine static cold storage have been summarized in numerous papers [2,4,22]. For instance, Op den Dries et al. compared NMP and conventional SCS with respect to their effects on liver function and bile duct preservation in both DCD and non-DCD livers. Their study, which utilized a rat model, showed that NEVLP mitigates hepatic injury and provides better bile duct protection compared to SCS, especially in DCD livers [23]. A similar study by Kollmann et al. investigated the impact of both preservation methods on platelet aggregation and platelet-mediated sinusoidal endothelial cell (SEC) injury during liver transplantation. Their findings indicated that NEVLP is a useful strategy for reducing platelet-mediated ischemia-reperfusion injury (IRI) during and after LT [24]. Unfortunately, these studies were exclusively based on biochemical panels, and there continues to be a lack of data in the literature regarding how preservation methods affect low-molecular-weight compounds in body fluids. This is a notable gap, as such information could shed new light on the metabolic processes in the organ. In comparing the composition of bile produced by the liver after NEVLP and SCS, we observed changes in the concentrations of some bile acids during reperfusion. Specifically, increased concentrations of GHCA, TUDCA, coprocholic acid and CDCA acid were observed in the bile from the SCS-treated livers, while increased concentrations of TCA and taurohyocholic acid were detected in the bile from the livers preserved via NEVLP (Table 1 and Table 2). Previous studies have reported significantly elevated levels of GHCA and TUDCA in bile and serum samples from patients with liver disease and primary sclerosing cholangitis (PSC) [25], as well as increased serum TUDCA levels in patients with primary biliary cirrhosis (PBC) [26]. TUDCA is a hydrophilic bile acid that protects against cholestatic and hepatocellular injury by enhancing the secretory capacity of the cholestatic liver cells and through its cytoprotective action against hydrophobic bile salts. In summarizing ischemia-reperfusion injury and ischemic-type biliary lesions following liver transplantation, Cursio and Gugenheim note that cytokines secreted by Kuppfer cells in the rejected organ contribute to the loss of Na (+), K (+)-ATPase activity, which plays an important role in bile secretion. Reduced activity of Na (+), K(+)-ATPase, a cotransporter for hepatocyte taurocholate uptake, leads to the impairment of TUDCA secretion and may result in subsequent cholestatic injury [27]. Our results did not reflect this assumption, as the bile samples from the SCS group were characterized by higher concentrations of TUDCA, which indicates that post-reperfusion graft injury after the use of SCS probably does not result from the presented mechanism. Moreover, increased concentrations of CDCA in biological materials have also been reported in liver diseases. For instance, Bathena et al. observed increased concentrations of CDCA in urine samples from patients suffering from various liver diseases, finding that levels of this bile acid were positively correlated with the stage of the disease [28]. Additionally, Mouzaki et al. found higher levels of CDCA in stool samples from non-alcoholic steatohepatitis (NASH) patients [29]. In the present study, the bile samples from the SCS group were characterized by a slightly higher concentration of CDCA compared to the NEVLP group and, as in the case of NASH patients, this finding may be due to the dominant role played by the alternative bile acid synthesis pathway. It has been suggested that the decreased expression of sterol 12-α-hydroxylase (CYP8B1) and the concomitant overexpression of oxysterol 7-α hydroxylase (CYP7B1) leads to increased CDCA synthesis, although the mechanism driving this relationship remains unclear [30]. In addition, arachidonic acid (AA) and 5-hydroxyeicosatetraenoic acid (5-HETE), two metabolites that may be associated with organ inflammation, were present in higher concentrations in bile samples collected from the SCS group during POD and reperfusion, respectively. AA is released from cell membranes by cytoplasmic phospholipase A2 alpha (cPLA2α), which can be activated during IRI [31,32]. Professor Kojima’s group demonstrated that AA suppresses the growth of hepatic cells by inducing the production of reactive oxygen species (ROS) and the activation of mediated cellular (mostly nuclear) transglutaminase 2 (TG2). The nuclear accumulation of TG2 induces hepatocyte apoptosis via Sp1 transcription factor cross-linking and inactivation, resulting in the inhibition of the c-Met expression required for hepatic cell viability [33,34]. In addition, arachidonate 5-lipoxygenase metabolizes AA to 5-hydroperoxyeicosatetraenoic acid, which is used to synthesize leukotrienes or is converted to 5-HETE with the release of ROS [31,35].

The analysis of how SCS and NEVLP affected the metabolic profile of bile further revealed differences in the levels of numerous lipids belonging to the LPC group (Table 1 and Table 2). Recent studies have found that changes associated with LPC 16:0 and 18:0 levels may play a role in signalling liver tissue damage and EAD in patients undergoing liver transplantation. For example, Xu et al. observed increased levels of these lipids in biopsy samples from patients with EAD [36], while Tsai et al. found lower levels of LPC 16:0 in plasma samples from patients with EAD [37]. It has been shown that LPCs can produce either pro- or anti-inflammatory effects. Pro-inflammatory effects, such as the expression of adhesion molecules, the release of chemotactic factors or an increase in ROS production, have mainly been attributed to saturated LPCs (LPC 16:0 and LPC 18:0) and monounsaturated LPC 18:1. On the other hand, polyunsaturated LPC species, such as LPC 22:4 and LPC 22:6, have been found to be anti-inflammatory and capable of neutralizing the in vivo inflammatory effect induced by saturated LPC 16:0 [38]. In our study, the bile samples from the SCS group were characterized by higher levels of saturated and monounsaturated LPCs compared to the NEVLP group, which suggests differences in the metabolic processes occurring in organs subjected to different preservation methods. Unfortunately, many of the processes occurring in the organ during the peri-transplant period—which in turn influence bile composition—remain unclear. Nonetheless, the metabolites identified in this comparison indicate that the type of preservation influences both the degree of damage to the bile ducts and the organ’s condition.

### 3.2. Effect of 30 Min Ischemia on the Metabolomic Profile of Bile after Transplantation

Analysis of the effect of short-term (30 min) graft ischemia on the composition of bile secreted after transplantation revealed that the most significant changes occur during the reperfusion of the organ following SCS (Tables S1 and S2). Notably, Op den Dries et al. obtained similar results in their comparison of non-DCD and DCD groups subjected to both preservation methods in rat models. In addition to an increase in markers of biliary epithelial cell injury (LDH and GGT) in the bile samples from the SCS group, Op den Dries et al. observed that ultrastructural changes were most prominent in the SCS-preserved DCD livers after reperfusion [23]. Some of the metabolites identified in our study have already been reported in previous studies as being associated with lesions in the organ. Patterson et al. found increased levels of oxidized bile acid in the plasma of HCC patients compared to a cancer group without hepatic involvement, as well as decreased LPC levels compared to a group of healthy volunteers [39]. Although relatively little is known about fetal 3-oxo-4,6-choladienoic acid in adults, increased levels of this compound have been observed in plasma samples from cirrhosis patients, which may be related to deterioration in the activity of Δ4-3-oxo-steroid 5β-reductase [40]. Moreover, it has been shown that 5-methoxyindoleacetic acid, produced by Lactobacillus sp., is transported from the intestine to the liver, where it participates in activating nuclear factor erythroid 2-related factor, which helps to protect against oxidative damage to the liver [41].

### 3.3. Influence of Degree of Ischemia on the Bile Metabolome in the Peri-Transplant Period during NEVLP

The analysis of ischemia’s impact on the function of livers subjected to NEVLP showed that 90 min of ischemia resulted in the most metabolic changes in the organ, which accounts for the differences in the bile’s metabolomic profile compared to the HBD group (Table 3). As indicated above, compared to NEVLP, the use of SCS resulted in increased LPC levels in the bile samples acquired during reperfusion. However, the levels of LPCs were observed to decrease in the case of worsening ischemia (Figure 5). As previously mentioned, Tsai et al. found that reduced levels of LPC lipids in plasma correlate with the occurrence of EAD in patients [42]. Furthermore, Tanaka et al. demonstrated a decrease in LPC levels in serum samples in a mouse NASH model and the disruption of bile acid homeostasis manifested by an increase in tauro-β-muricholate and TCA levels. Differences in LPC levels can be attributed to changes in the activity of lysophosphatidylcholine acyltransferases (LPCATs) in converting LPC to PC, while differences in bile acid homeostasis may be due to the influence of inflammation on the classical and alternative pathways of its synthesis [43].

Although this study yielded promising results, it has some limitations. First, the analysis in this work focused exclusively on bile samples. To better understand the mechanisms driving the observed metabolic changes, it would be helpful to simultaneously analyze the graft tissue and blood samples as well. Another limitation is that the obtained results were not compared with routinely assessed clinical parameters; such a comparison would help to ensure a comprehensive evaluation of the results. Moreover, the analysis presented herein was non-targeted, which means that it requires confirmation via a fully validated targeted method. Given these limitations, subsequent experiments have already been planned to simultaneously analyze tissue, bile, perfusate and blood samples from liver patients in order to develop targeted approaches for potential biomarkers.

## 4. Materials and Methods

### 4.1. Animals

Male Yorkshire pigs weighing 29–35 kg were used for this study. The experiments in this work were approved by the Animal Resource Centre from University Health Network. All animals used in this work received humane care in accordance with the “Guide for the Care of Laboratory Animals” published by the National Institutes of Health.

### 4.2. Study Design

**Figure 7:**
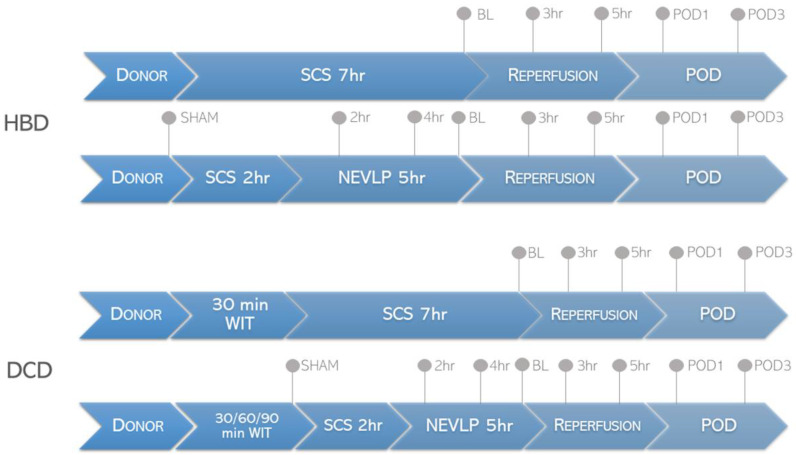
Experimental groups and design. Prior to procurement, animals were subjected to 0 min (HBD; heart beating donors), 30 min, 60 min and 90 min of warm ischemia to mimic donation-after cardiac death (DCD). Gray dots represent bile sampling time points. SHAM—prior to organ harvest; BL—baseline (start of reperfusion); WIT—warm ischemia time.

The analyses were performed on bile samples from porcine model donors with mild (heart beating donor [HBD]) and moderate warm ischemia (donation after circulatory death [DCD]) grafts. The obtained livers were subjected to 7 h SCS or NEVLP before transplantation. The SCS group consisted of two subgroups (5 animals in each group): HBD (HBD-SCS) livers and DCD livers with 30 min ischemia time (30′DCD-SCS). The livers from the NEVLP groups (HBD/30′DCD/60′DCD/90′DCD-NEVLP (5 animals in each group)) were stored at 4 °C in histidine-tryptophan-ketoglutarate (HTK) solution during the back-table preparation for ex vivo perfusion and were subsequently subjected to 5 h NEVLP at 37 °C. The livers from the SCS and NEVLP groups were subjected to a preservation time of 7 h, followed by transplantation into the recipient pigs. The recipient pigs were followed for a survival period of 4 days. Bile samples were collected during the peri-transplant period at the time points shown in Figure 7. The research material was provided by scientists from the Department of Surgery at the Toronto General Hospital (University Health Network, Toronto, ON, Canada).

The HBD pigs received heparin at 500 international units/kg of body weight 5 min prior to cross-clamping and cold flushing. In the case of the DCD grafts, the donor pigs received the same dose of heparin 5 min prior to the induction of cardiac arrest, which was accomplished by the intracardiac infusion of potassium chloride (20 mEq). After the induction of cardiac arrest, the desired warm ischemia time was awaited according to the protocol for the respective DCD model (30 min, 60 min or 90 min). Subsequently, all livers were flushed with a total volume of 3 L cold (4 °C) Custodiol-HTK (Essential Pharmaceuticals, LCC, Ewing, NJ, USA) through the aorta and portal vein. In the SCS groups, the livers were packed in bags filled with Custodiol-HTK and then stored in an icebox (4 °C) for 7 h; in the NEVLP-groups, the livers were cannulated and prepared for perfusion on ice (4 °C). To ensure the preservation time was comparable for all experiments, livers from the NEVLP groups were stored on ice for 2 h before being perfused for 5 h at 37 °C. After 5 h of NEVLP, the livers were flushed with cold (4 °C) Custodiol-HTK and then stored on ice before implantation was performed. Following SCS and NEVLP, the grafts were transplanted into recipient pigs using the method described in [44,45]. Animals were euthanized under deep anesthesia on postoperative day 4.

### 4.3. Normothermic Ex Vivo Liver Perfusion Setup

The ex vivio perfusion circuit was composed of a centrifugal pump (Rotaflow centrifugal pump), 2 hard shell reservoirs (Maquet, Hirrlingen, Germany), a leukocyte filter and a hollow-fiber dialyzer (NR16, Fresenius, Bad Homburg, Germany). The setup was similar to the OrganOx Metra that has recently been published in human clinical trials and has been described previously [46,47,48]. The hepatic artery pressure was set to 50–60 mm Hg, resulting in a flow of up to 400 mL/h. A second reservoir was used to regulate the portal vein pressure, which was intended to reach 2–4 mm Hg and a flow of 900–1400 mL/h. Porcine blood was obtained from the donor animal shortly before liver retrieval and erythrocytes were passed through leukocyte filters. For the perfusate, 1.5 L of Steen Solution (XVIVO Perfusion, Goteborg, Sweden) was mixed with the washed porcine erythrocytes (around 400 g) to achieve a final hematocrit of 15% with a hemoglobin level of 45 mg/dL. During the priming of the circuit, Heparin (10,000 international units (IU), Sandoz Canada, Quebec, QC, Canada), amino acid concentrate (Travasol 4.25%, 50 mL Bolus, Baxter, Hamilton, ON, Canada), sodium bicarbonate (20 mmol), calcium chloride (9.2 mmol/L) and antibiotics (Cefazolin—1 g, Pharmaceutical Partners of Canada, Richmond Hill, ON, Canada and Metronidazole—500 mg, Baxter, Mississauga, ON, Canada) were added. Additionally, amino acid concentrate (Travasol 4.25%, 8 mL/h, Baxter, Hamilton, ON, Canada), insulin (125 IU/h, NovoRapid, Novo Nordisk, Mississauga, ON, Canada), 2% taurocholic acid (7 mL/h, Sigma-Aldrich, St. Louis, MO, USA infused as a precursor for bile production) and prostaglandin E1 (500 μg/3 h, Pfizer, Kirkland, QC, Canada) were continuously administered during the perfusion. The surgical protocol for the donor liver retrieval and the liver transplantation has been described in more detail previously [24,44,45].

### 4.4. Sample Preparation

LC-MS-grade water, methanol, acetonitrile and formic acid were purchased from Merck (Poznań, Poland). The phosphate-buffered saline solution (PBS; pH 7.4) was prepared using analytical-grade sodium chloride, potassium chloride, potassium phosphate monobasic, sodium phosphate dibasic, hydrochloric acid and sodium hydroxide obtained from Merck (Poznań, Poland).

Sample preparation was performed via SPME, with each step of the process being performed on a high-throughput 96-manual thin-film microextraction (TFME) system (Professional Analytical System (PAS) Technology, Magdala, Germany). The use of this system allowed all of the samples to be analyzed simultaneously [9]. Extractions were performed using steel blades coated with a Hydrophilic-lipophilic balanced (HLB) N-vinylpyrrolidone-divinylbenzene copolymer sorbent (Alchem, Toruń, Poland). The coating preparation procedure was based on the spraying method described by Mirnaghi et al. [49]. The steel blades were purchased form Professional Analytical System (PAS) Technology (Magdala, Germany), while the polypropylene Nunc 96 DeepWell plates were purchased from Merck (Poznań, Poland).

Before beginning the extractions, the SPME blades were conditioned for 30 min in 1.0 mL of methanol:water (50:50; *v*/*v*) solution in 96-well-plates with agitation at 1000 rpm. After conditioning, a 10 s wash step was performed. Extractions were performed from 1.0 mL of diluted bile (1:99 in PBS) with the addition of glycodeoxycholic-2,2,4,4-d_4_ acid (Merck, Poland) as an internal standard for 1 h (1000 rpm). After extraction, the blades were placed in 1 mL of nanopure water for 10 s. Following the wash step, desorption was conducted in 1 mL of ACN:H_2_O (80:20; *v*/*v*) with agitation (1000 rpm) for 2 h.

### 4.5. LC-MS Analysis

Chromatographic separation was performed on a Dionex UHPLC system. Bile extracts, obtained per the method described above, were injected at a volume of 10 µL on a reversed-phase pentafluorophenyl (PFP) column (Discovery HS F5 100 × 2.1 mm, 3 μm). The autosampler and column temperatures were set to 4 °C and 25 °C, respectively, and the flow rate was set to 0.3 mL/min. Mobile phase A consisted of water with formic acid (99.9:0.1; *v*/*v*) and mobile phase B comprised acetonitrile and formic acid (99.9:0.1; *v*/*v*). The total analysis time for each sample was 40 min. The starting mobile phase conditions were as follows: 0% B from 0 to 3.0 min; a linear gradient to 90% B from 3.0 to 25.0 min; an isocratic hold at 90% B from 25.0 min to 34.0 min; and finally, a 6 min column re-equilibration time [50].

The analyses were performed in both positive and negative electrospray ionisation modes in separate runs on a Q Exactive Focus Orbitrap mass spectrometer (Thermo Fisher Scientific, Bremen, Germany). In positive ionization mode, the following HESI ion source parameters were used: spray voltage—1500 V; capillary temperature—300 °C; sheath gas—40 a.u.; aux gas flow rate—15 a.u.; probe heater temperature—300 °C; and S-Lens RF level—55%. For negative ionization mode, the HESI ion source parameters were as follows: spray voltage—2500 V; capillary temperature—256 °C; sheath gas—48 a.u.; aux gas flow rate—11 a.u.; probe heater temperature—413 °C; and S-Lens RF level—55%. The scan range was set to m/z 80–1000 with a resolution of 70,000. The instrument was calibrated using external calibration immediately before the analysis and every 48 h thereafter, resulting in a mass accuracy of <2 ppm. Data acquisition was performed using Xcalibur software v. 4.2 and Free Style 1.4 (Thermo Fisher Scientific, San Jose, CA, USA).

All samples were analysed in one randomised sequence and QC samples were run periodically (8–10 injections) to verify the instrument’s performance. QC samples were prepared by mixing 20 μL of each of the bile extracts.

The putative identification of compounds was confirmed based on Full MS/dd-MS2 mode. The fragmentation parameters were as follows: mass resolution—35,000 full width at half maximum (FWHM); AGC target—2 × 10^4^; minimum AGC—8 × 10^3^; intensity threshold—auto; maximum IT—auto; isolation window—3.0 m/z; stepped collision energy—10 V, 20 V, 40 V; loop count—2; and dynamic exclusion—auto. Fragmentation spectra were confirmed using online databases such as LIPID MAPS, HMDB, METLIN and mzCloud.

### 4.6. Data Processing and Statistical Analysis

The raw data were processed using Compound Discoverer 3.1 (Thermo Fisher Scientific, San Jose, CA, USA) software, with the aim of identifying metabolites present in the samples. Detected metabolites with a signal-to-noise ratio > 3 and a peak intensity > 100,000 were subjected to further analysis. The intensity tolerance was set at 30%, and the RT tolerance was set at 0.2 min. The QC-based area was used for correction (min 80% coverage, max 30% RSD in QC). After peak alignment, gap filling was applied to fill in the missing values via a very small peak at the level of spectrum noise for the compound. The effect of the extraction process on the results was normalized by internal standard. After confirming the identification of the compounds, the results were summarized in a table and entered into MetaboAnalyst 5.0 software (accessed on 25 November 2022). Data were normalized by median, log-transformation and Pareto scaling. PCA was used to visually assess the separation between the sample groups. The differences among groups were evaluated using the Kruskal—Wallis test and multiple (post-hoc) mean-ranks comparisons using Statistica 13.3 PL software (StatSoft, Inc., Tulsa, OK, USA). Differences were deemed to be significant if *p* < 0.05. If only two groups were compared, the results were illustrated using a Volcano plot, which combines results from Fold Change (FC > 2) analysis and Wilcoxon rank-sum test (*p* < 0.05) with FDR correction into one single graph. Partial least squares discriminant analysis (PLS-DA) was used to assess the separation between groups and the model was cross-validated using leave-one-out cross validation. Finally, a pattern-matching method based on Spearman’s rank correlation coefficient was implemented to search for compounds present at levels that were linearly correlated with organ ischemia time.

## 5. Conclusions

This study had two main goals: to evaluate how different preservation methods and ischemia impact the metabolome of bile produced by livers in the peri-transplant period, and to identify compounds that can be used as indicators of organ quality. To this end, high-throughput sample-analysis protocol based on TFME was developed and successfully applied to prepare bile samples—a matrix with a high content of lipid components and high variability with respect to its physicochemical properties. The analysis of the impact of preservation methods revealed that the first changes in the bile metabolome appeared only during the ongoing reperfusion. Bile produced in the SCS-preserved livers was characterized by increased levels of metabolites such as CDCA, AA and 5S-HETE, as well as saturated and monounsaturated LPCs. Such changes may be associated with differences in the bile acid synthesis pathways and organ inflammation. The study showed that short ischemia of the organ prior to bile collection led to changes in the metabolome of the bile produced by SCS-preserved organs during reperfusion. In contrast, the use of NEVLP reduced the negative effect of ischemia on organ function, as significant changes in bile composition were only visible after 90 min of ischemia. Notably, a linear relationship was observed between levels of lipids from the LPC group and the time of organ ischemia. Thus, this study identified metabolites worth considering as potential markers of changes occurring in preserved grafts in future analyses of larger study groups.

## Figures and Tables

**Table 1:** Statistically significant compounds identified in SCS- and NEVLP-preserved organs from HBD and 30′DCD donors at particular time points. Data were acquired in positive ionization mode. The Kruskal—Wallis test and multiple (post-hoc) mean-rank comparisons were used to select the metabolites statistically significantly different from the SCS and NEVLP methods. Results are presented as fold change.

Name	Molecular Weight	RT	The Effect of the Organ Preservation Method (SCS vs. NEVLP)					
			Baseline (*n* = 5)		Reperfusion (*n* = 10)		POD (*n* = 10)	
			$\frac{HBDSCS}{HBDNEVLP}$	$\frac{30^{\prime }DCDSCS}{30^{\prime }DCDNEVLP}$	$\frac{HBDSCS}{HBDNEVLP}$	$\frac{30^{\prime }DCDSCS}{30^{\prime }DCDNEVLP}$	$\frac{HBDSCS}{HBDNEVLP}$	$\frac{30^{\prime }DCDSCS}{30^{\prime }DCDNEVLP}$
			FC	FC	FC	FC	FC	FC
Taurine	125.01	10.71	−1.18	2.19	−3.13 *	−1.19	−1.35	−1.39
4-Methylene-2-pyrrolidinecarboxylic acid	127.06	7.26	−2.50	1.09	−2.00 *	−1.75	−2.50	1.20
Pyroglutamic acid	129.04	1.50	−2.94	1.01	−2.5 *	−1.25	−1.61	1.75
Leucine/Isoleucine	131.09	8.13	−1.19	−1.11	1.33	1.73 *	1.18	−1.69
1H-Indole-3-carboxaldehyde	145.05	11.26	1.16	1.01	−1.52 *	1.16	1.01	−1.08
Tryptophan	204.09	11.25	1.14	−1.04	−7.14 **	−2.86	1.06	−1.10
2,5-Dichloro-4-oxohex-2-enedioate	225.94	1.96	1.02	−1.14	−1.37 **	1.10	−1.35 *	−1.49
11-hydroxy-9-tridecenoic acid	228.17	14.80	1.05	−1.43	−1.69 **	−1.11	−1.43	−1.85
N-Acetyl-tryptophan	246.10	11.26	−1.12	−1.16	−10.00 **	−4.76	−1.04	−1.14
4,9-heptadeca-1,4,9-trien-6-yn-3-ol	246.20	20.46	1.18	−1.39	−1.41	−1.08	−1.20	−2.27 **
Asn-Arg	288.16	8.72	−1.16	−1.39	−1.52 **	−1.04	−1.25	−2.00
3-keto stearic acid	298.25	19.49	−1.10	−1.35	−1.47 *	−1.15	−1.30	−2.13
Arachidonic acid	304.24	21.14	1.90	1.14	2.12 *	1.27	4.15	−1.25
methyl 9,10-epoxy-12,15-octadecadienoate	308.24	20.99	−3.23	−1.19	−3.13 *	−1.52	−1.49	−4.76
1-Acetoxy-2-hydroxy-16-heptadecyn-4-one	324.23	18.22	−1.18	−1.37	−1.09	2.08 *	1.04	−1.22
3-hydroxy-4-isopropylbenzyl alcohol 3-glucoside	328.15	21.03	−2.22	−1.43	−2.56 **	−1.22	−1.02	−1.96
Docosapentaenoic acid methyl ester	344.27	15.41	1.66	−1.52	1.98 *	−1.32	1.04	−1.28
Tetracosahexaenoic acid	356.27	14.69	1.78	−1.03	−1.20	−2.78 *	−1.19	−1.28
Docosahexenoyl-EA	371.28	13.55	−1.59	1.23	−11.11 **	−7.14 **	−2.70 **	−1.18
Leu-Asn-His (LNH)	382.20	19.77	1.25	−1.52	−1.52 *	1.00	−1.15	−1.79
9′-Carboxy-alpha-chromanol	390.28	15.78	1.69	1.71	2.46	1.34	12.83 *	−1.75
22-Dehydroclerosterol or Stigmasta-7,22,25-trien-3-ol	410.36	18.93	1.02	−1.10	−2.00	−3.57 **	−1.64	−1.12
Tricosanoylglycine	411.37	22.62	1.04	−1.56	−1.56 *	−1.15	−1.49	−2.13 *
LPE 14:0	425.26	13.98	−1.18	2.14	−1.28	1.65	−2.27	2.08 *
(3b,5a,6a,9a,22,24)-Ergosta-7,22-diene-3,5,6,9-tetrol	446.34	25.12	1.17	−1.61	−1.56 **	1.02	−1.28	−1.61
Glycoursodeoxycholic acid	449.31	13.44	1.65	1.75	1.21	2.25 *	1.99	2.48
Glycohyocholic acid	465.31	12.71	2.30	2.05	−1.01	4.46 **	−1.08	1.42
LPC 14:0	467.30	16.60	1.62	1.05	2.21 *	1.68	3.20 **	−1.1
LPC 15:0	481.32	17.18	1.18	−1.35	2.82 *	2.29	4.12 **	−1.54
LPC 16:1	493.32	17.00	1.89	−1.12	2.96 *	1.90	3.48 *	−1.23
LPC 16:0	495.33	17.74	1.61	−1.27	2.67 *	2.14	3.16 *	−1.69
Tauroursodeoxycholic acid	499.30	11.26	1.28	1.09	−1.05	1.86 *	1.20	1.17
LPE 20:4	501.28	17.92	1.56	−1.67	1.01	1.28	3.37 *	−1.69
PE 20:1	507.33	17.38	1.64	−1.54	2.16 *	1.71	4.42	−1.35
LPC 17:0	509.35	18.29	1.65	−1.43	2.30	1.97	5.23 **	−2.00
Sulfoglycolithocholic acid	513.28	10.21	−1.10	2.69	−4.35 *	−2.70	−1.67	1.49
Taurocholic acid	515.29	10.84	−2.38	1.47	−50.00 **	−25.00 *	−4.35 *	−1.49
Taurohyocholic acid	515.29	10.71	−1.30	1.68	−4.76 **	−1.56	−1.32	−1.33
Tauro-b-murocholic acid	515.29	10.28	3.06	1.23	−2.70	3.56 *	−1.75	−2.00
LPC 18:0	523.37	18.84	1.53	−1.30	2.20 *	1.86	3.13 *	−2.22
LPS 20:4	545.28	16.28	4.56	1.21	2.42	3.07 *	−1.25	1.01
LPC 22:1	577.41	18.15	1.23	−1.19	1.15	2.29 *	1.88	6.90
SM 16:1;O2/18:0	702.57	22.64	1.19	1.20	−1.64 *	−1.59	1.26	1.05
PC 32:2	729.53	24.01	−1.04	−1.12	−1.79 *	1.01	−1.12	−1.08
PE 36:4	741.53	24.23	−1.54	1.57	−1.82 *	−1.02	−1.41	1.30
PE 38:4	767.55	24.94	−1.75	1.21	−1.75 *	−1.28	−1.23	1.10

− denotes an increased level in the group located in the denominator of the fraction; FC—fold change; *—*p* < 0.05; **—*p* < 0.01.

**Table 2:** Statistically significant compounds identified in SCS- and NEVLP-preserved organs from HBD and 30′DCD donors at particular time points. Data were acquired in negative ionization mode. The Kruskal—Wallis test and multiple (post-hoc) mean-rank comparisons were used to select the metabolites statistically significantly different from the SCS and NEVLP methods. Results are presented as fold change.

Name	Molecular Weight	RT	The Effect of the Organ Preservation Method (SCS vs. NEVLP)					
			Baseline (*n* = 5)		Reperfusion (*n* = 10)		POD (*n* = 10)	
			$\frac{HBDSCS}{HBDNEVLP}$	$\frac{30^{\prime }DCDSCS}{30^{\prime }DCDNEVLP}$	$\frac{HBDSCS}{HBDNEVLP}$	$\frac{30^{\prime }DCDSCS}{30^{\prime }DCDNEVLP}$	$\frac{HBDSCS}{HBDNEVLP}$	$\frac{30^{\prime }DCDSCS}{30^{\prime }DCDNEVLP}$
			FC	FC	FC	FC	FC	FC
Glucuronic acid	194.04	1.17	−2.70	1.35	−2.44 *	−1.10	−5.56	3.61
Cinnamoylglycine	205.07	11.46	1.79	1.19	−1.14	−2.04 *	1.56	1.58
Indolelactic acid	205.07	13.65	−1.82	1.14	−1.85 *	−1.56	−1.85	1.43
Ribose-5-phosphate	230.02	1.17	−2.70 *	1.27	−2.17 *	−1.12	−4.55	2.84
Tridecanedioic acid	244.17	15.26	1.30	−1.47	−1.37	1.03	1.00	−2.22 *
N-Acetyl-tryptophan	246.10	11.23	1.32	1.00	−5.56 **	−2.17	1.46	1.37
5S-HETE	320.24	18.94	8.33	−2.04	11.36 **	2.35	2.04	−5.56
Chenodeoxycholic acid	392.29	16.28	2.08	1.13	1.86	1.04	3.24 *	−5.00
N-Palmitoyl tyrosine	419.30	15.97	6.13	−1.23	7.33 **	−2.08	5.38	−4.17
Glycoursodeoxycholic acid	449.31	13.44	1.50	1.50	1.28	1.80 *	2.52	2.72
3a,7a,12a-trihydroxy-5b-cholestan-26-oic acid	450.34	16.06	2.16	2.36	1.94 *	2.03 *	−1.96	−1.59
Glycocholic acid	465.31	13.19	−1.03	1.68	−1.30	1.99 *	−1.28	−1.54
Cholesterol sulfate	466.31	12.49	2.07	1.24	−1.75	2.77 *	−1.30	1.03
Retinoyl b-glucuronide	476.24	16.60	23.25	−1.18	6.30 *	−2.13	−1.72	3.50 *
1-O-all-trans-retinoyl-beta-glucuronic acid	476.24	16.94	3.97	−1.45	5.36	1.27	−2.78	5.50 *
LPE 18:0	481.32	18.62	1.38	−2.63	1.93	2.02	3.90 *	−2.00
4-Hydroxyretinoic acid glucuronide	492.24	13.62	13.7	1.45	9.45 **	−2.44	−1.92	2.30
LPC 17:1	507.33	18.11	1.61 *	−1.67	1.20	1.46	3.02	−1.10
LPC 17:0	509.35	18.29	1.75	−1.20	2.84	2.47	3.85 *	−2.50
Taurohyocholic acid	515.29	10.71	1.27	2.65	−5.26 *	−1.89	−1.15	−1.39
Taurocholic acid	515.29	10.84	−1.30	1.75	−50.00 **	−20.00 *	−2.63	−1.43
LPS 20:4	545.28	16.29	5.10	1.07	2.56 *	4.57	−1.02	1.03

− denotes an increased level in the group located in the denominator of the fraction; FC—fold change; *—*p* < 0.05; **—*p* < 0.01.

**Table 3:** Number of metabolites (pos/neg) differentiating the moderated ischemia (30′DCD, 60′DCD and 90′DCD) groups from the HBD group at different phases of the peri-transplant period. The number of metabolites exhibiting significant changes was determined based on a Volcano plot (fold change > 2, *p* < 0.05 with FDR correction). ↓ indicates down-regulation and ↑ indicates up-regulation after ischemia.

	Perfusion (*n* = 5)		Reperfusion (*n* = 10)		POD (*n* = 10)	
	↑	↓	↑	↓	↑	↓
30′DCD vs. HBD	0/1	0/0	0/0	0/0	1/0	0/0
60′DCD vs. HBD	12/5	12/9	4/1	11/2	0/0	5/0
90′DCD vs. HBD	44/17	26/10	22/11	21/19	6/2	18/5

## Data Availability

Not applicable.

## Supplementary Materials

The following supporting information can be downloaded at: https://www.mdpi.com/article/10.3390/ijms24032127/s1.
