# Supplementary material for: Biliary Metabolome Profiling for Evaluation of Liver Metabolism and Biliary Tract Function Related to Organ Preservation Method and Degree of Ischemia in a Porcine Model
Source: Int J Mol Sci. 2023 Jan 21;24(3):2127. doi: 10.3390/ijms24032127 (PMC9916698; doi:10.3390/ijms24032127)
Supplement: Supplementary file 1 [file ijms-24-02127-s001.zip › ijms-2098382-supplementary.pdf]

Biliary Metabolome Profiling for Evaluation of Liver Metabolism and Biliary Tract Function Related to Organ Preservation Method and Degree of Ischemia in a Porcine Model

Kamil Łuczykowski [1], Natalia Warmuzińska [1], Dagmar Kollmann [2,3], Markus Selzner [2], Barbara Bojko [1,*]

[1] Department of Pharmacodynamics and Molecular Pharmacology, Faculty of Pharmacy, Nicolaus Copernicus University in Toruń, Collegium Medicum in Bydgoszcz, Bydgoszcz, Poland
[2] Department of Surgery, Ajmera Transplant Centre, Toronto General Hospital, University Health Network, Toronto, Canada
[3] Department of General Surgery, Medical University of Vienna, Vienna, Austria
* Correspondence: bbojko@cm.umk.pl

Supplementary Material

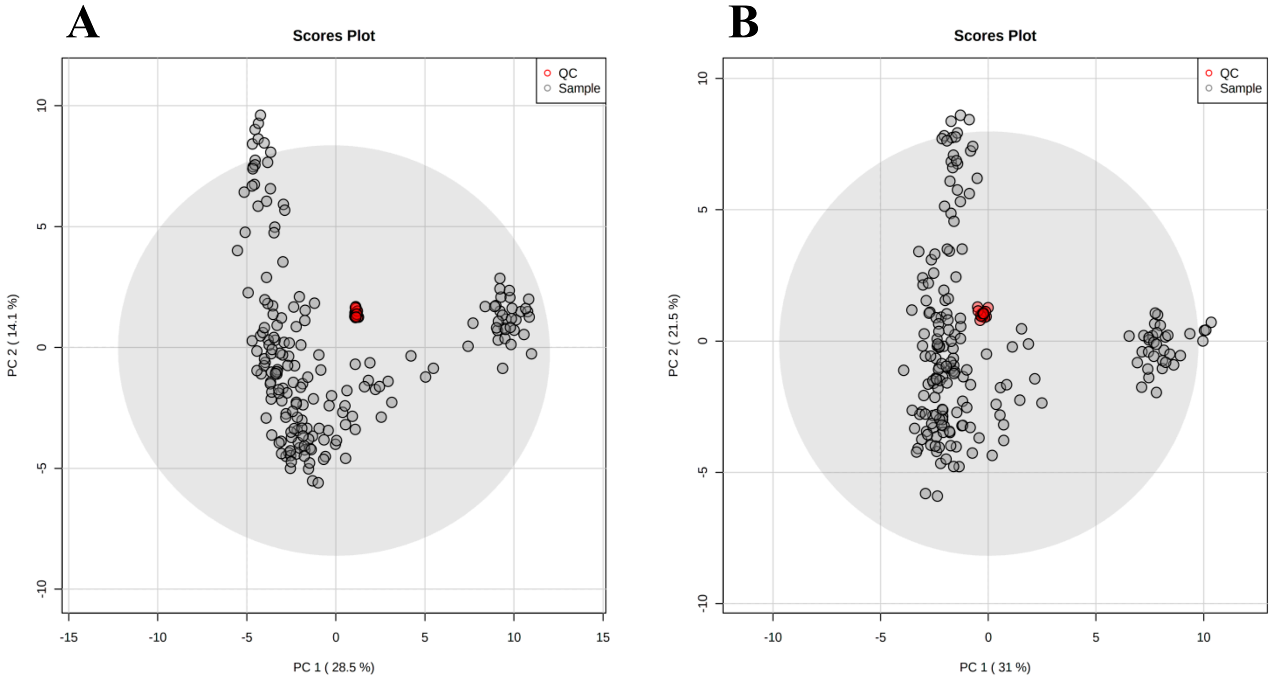

Figure S1. Principal component analysis (PCA) plots of all analyzed samples and extraction quality control (QC) samples for positive ionization mode (A) and negative ionization mode (B)

Table S1. The list of statistically significant compounds in comparison HBD versus 30'DCD for SCS and NEVLP donors in particular time points. Data for positive ionization mode.

Name	Molecular weight	RT	Effect of 30-minute ischemia (HBD vs 30'DCD)					
			Baseline (n=5)		Reperfusion (n=10)		POD (n=10)	
			HBD SCS / 30′DCD SCS	HBD NEVLP / 30′DCD NEVLP	HBD SCS / 30′DCD SCS	HBD NEVLP / 30′DCD NEVLP	HBD SCS / 30′DCD SCS	HBD NEVLP / 30′DCD NEVLP
			FC	FC	FC	FC	FC	FC
Indole	117.06	13.82	-1.25	-1.32	**-1.89****	1.02	-1.12	1.04
5-amino-pentanoic acid	117.08	1.63	-1.25	-1.49	**-2.08****	1.05	-1.19	-1.69
4-Methylene-2-pyrrolidinecarboxylic acid	127.06	7.26	**-3.45***	-1.25	-1.41	-1.23	-3.23	-1.05
Pyroglutamic acid	129.04	1.50	-2.70	1.08	**-2.22***	-1.11	**-2.63***	1.06
1H-Indole-3-carboxaldehyde	145.05	11.26	-1.11	-1.28	**-1.54***	1.15	-1.08	-1.16
Phenylalanine	165.08	9.43	-1.59	-1.25	**-1.64***	1.00	-1.52	-2.22
2,5-Dichloro-4-oxohex-2-enedioate	225.94	1.96	-1.19	-1.39	**-1.33***	1.14	-1.05	-1.18
Palmitamide	255.26	20.25	-1.25	-1.79	**-1.39***	-1.09	-1.20	-1.72
Linoleamide	279.26	19.88	-1.08	1.01	**-2.17***	-1.47	-2.17	-3.57
Stearamide	283.29	21.38	-1.28	-1.67	**-1.47****	-1.02	-1.30	-1.85
N-isobutyl-2,4-octadecadienoyl amine	335.32	21.89	-1.39	-1.64	**-2.00***	-1.39	-1.59	-1.92
Docosanamide	339.35	23.35	-1.39	-1.69	**-1.49***	-1.15	-1.32	-1.61
Tetrahydrocorticosterone	350.25	15.99	2.29	1.66	**2.37***	2.55	-1.06	1.45
3-Oxo-4,6-choladienoic acid	370.25	16.08	-7.69	1.95	-2.17	**-2.94***	-1.96	-1.10
12-Oxochenodeoxycholic acid	406.27	13.99	-2.44	-1.06	**-3.33****	**-1.54***	-1.75	-1.09

N-Docosahexaenoyl GABA	413.29	13.82	-1.3	-1.32	**-1.85****	1.08	-1.04	1.03
LPE 14:0	425.26	13.98	-1.85	1.36	-1.79	1.19	**-2.22***	**2.14***
Glycoursodeoxycholic acid	449.31	13.44	-1.23	-1.18	**-2.38****	-1.28	-1.22	1.02
Glycocholic acid	465.31	13.20	-1.08	1.19	**-1.61***	1.44	-1.05	-1.03
Glycohyocholic acid	465.31	12.71	1.55	1.38	**-2.44***	1.86	-1.27	1.21
PE O-18:0	481.32	18.62	2.11	-2.04	1.03	1.07	**4.36***	-1.75
LPC 15:0	481.32	17.18	1.75	1.10	1.47	1.19	**3.41***	-1.85
LPC 17:0	509.35	18.29	2.29	-1.03	1.60	1.37	**5.50***	-1.89
LPC 18:2	519.33	17.29	1.52	-1.10	**1.76****	1.81	1.77	1.29
LPC 18:0	523.37	18.84	1.72	-1.16	1.27	1.08	**3.34***	-2.08
PC 32:2	729.53	24.01	1.24	1.15	-1.01	**1.79***	1.52	1.58
PC 32:1	731.55	24.90	1.43	-1.59	-1.04	**1.62***	1.39	1.31
PE 36:4	741.53	24.23	-1.61	1.49	**-1.82***	-1.01	1.07	1.98

- denotes an increased level in the group located in the denominator of the fraction; *- p<0,05; **- p<0.01

Table S2. The list of statistically significant compounds in comparison HBD versus 30'DCD for SCS and NEVLP donors in particular time points. Data for negative ionization mode.

Name	Molecular weight	RT	Effect of 30-minute ischemia (HBD vs 30'DCD)					
			Baseline (n=5)		Reperfusion (n=10)		POD (n=10)	
			HBD SCS / 30'DCD SCS	HBD NEVLP / 30'DCD NEVLP	HBD SCS / 30'DCD SCS	HBD NEVLP / 30'DCD NEVLP	HBD SCS / 30'DCD SCS	HBD NEVLP / 30'DCD NEVLP
			FC	FC	FC	FC	FC	FC
Glucuronic acid	194.04	1.17	**-3.03***	1.21	-2.38	-1.05	-11.11	1.76
5-Methoxyindoleacetic acid	205.07	14.18	**-2.7****	1.22	-1.16	-1.06	-2.00	1.13
Ribose-5-phosphate	230.02	1.17	**-2.86***	1.21	-1.96	-1.01	-7.69	1.64
Tridecanedioic acid	244.17	15.26	1.01	-1.89	-1.32	1.08	-1.01	**-2.27***
5S-HETE	320.24	18.94	**9.46***	-1.79	2.15	-2.27	2.41	-4.55
N-Palmitoyl tyrosine	419.30	15.97	6.28	-1.20	**5.00***	-3.13	5.74	-4.00
Cortisol 21-sulfate	442.17	9.20	-1.61	-1.01	**2.74***	-1.12	-1.33	-1.05
Retinoyl b-glucuronide	476.24	16.60	9.68	-2.78	5.06	-2.63	-1.12	**5.34***
1-O-all-trans-retinoyl-beta-glucuronic acid	476.24	16.94	4.43	-1.30	4.70	1.12	-2.22	**6.94****
alpha-Ionol O-[arabinosyl-(1->6)-glucoside]	488.26	14.05	2.31	1.70	**5.41***	2.76	-2.04	1.80
4-Hydroxyretinoic acid glucuronide	492.24	13.62	6.29	-1.49	6.50	**-3.45***	-1.30	3.39
N-[(3a.5b.7a.12a)-3.7-dihydroxy-24-oxo-12-(sulfooxy)cholan-24-yl]-Glycine	545.27	10.62	1.08	1.48	-2.17	1.28	**-2.78****	-1.20

- denotes an increased level in the group located in the denominator of the fraction; *- p<0.05; **- p<0.01

Table S3. Detailed data of linear correlation coefficients and significance levels for metabolite correlated with organ ischemia.

Name	Molecular Weight	RT [min]	Correlation	Raw p-value	FDR adjusted p-value
		**Positive ionization mode**			
PE P-18:0	481.32	18.62	-0.80659	1.7404e-05	0.0013468
LPC 18:3	517.32	16.92	-0.79883	2.405e-05	0.0013468
LPC 17:1	507.33	17.56	-0.79883	2.405e-05	0.0013468
LPE 18:2	477.29	17.29	-0.78332	4.4148e-05	0.0018542
PE 20:1	507.33	17.38	-0.77557	5.8753e-05	0.0018563
LPC 20:4	543.33	17.56	-0.76781	7.7345e-05	0.0018563
LPC 22:6	567.33	17.64	-0.76781	7.7345e-05	0.0018563
LPC 16:1	493.32	17.00	-0.76006	0.00010079	0.0021166
LPC 16:0	495.33	17.74	-0.7523	0.0001301	0.0021857
LPC 14:0	467.30	16.60	-0.7523	0.0001301	0.0021857
LPC 18:0	523.37	18.84	-0.74454	0.00016644	0.002542
LPC 18:1	535.33	15.07	-0.72903	0.00026575	0.003189
LPC 18:2	519.33	17.29	-0.72903	0.00026575	0.003189
LPC 15:0	481.32	17.18	-0.72903	0.00026575	0.003189
Arachidonic acid	304.24	21.14	-0.72128	0.00033198	0.0037181
		**Negative ionization mode**			
5S-HETE	320.24	18.94	-0.85617	1.4664e-06	0.00012171
LPC 17:0	509.35	18.86	-0.82093	9.1997e-06	0.0002914
LPE 18:2	477,29	17.29	-0.81799	1.0533e-05	0.0002914
LPE 18:1	479,30	17,91	-0.80122	2.1807e-05	0.0004525
LPC 17:1	507.33	17.56	-0.79525	2.7795e-05	0.00046139
LPE 20:4	501,29	17,39	-0.73915	0.00019656	0.0027191
9,10,13-TriHOME	330,24	13,64	-0.7339	0.00023022	0.0027297

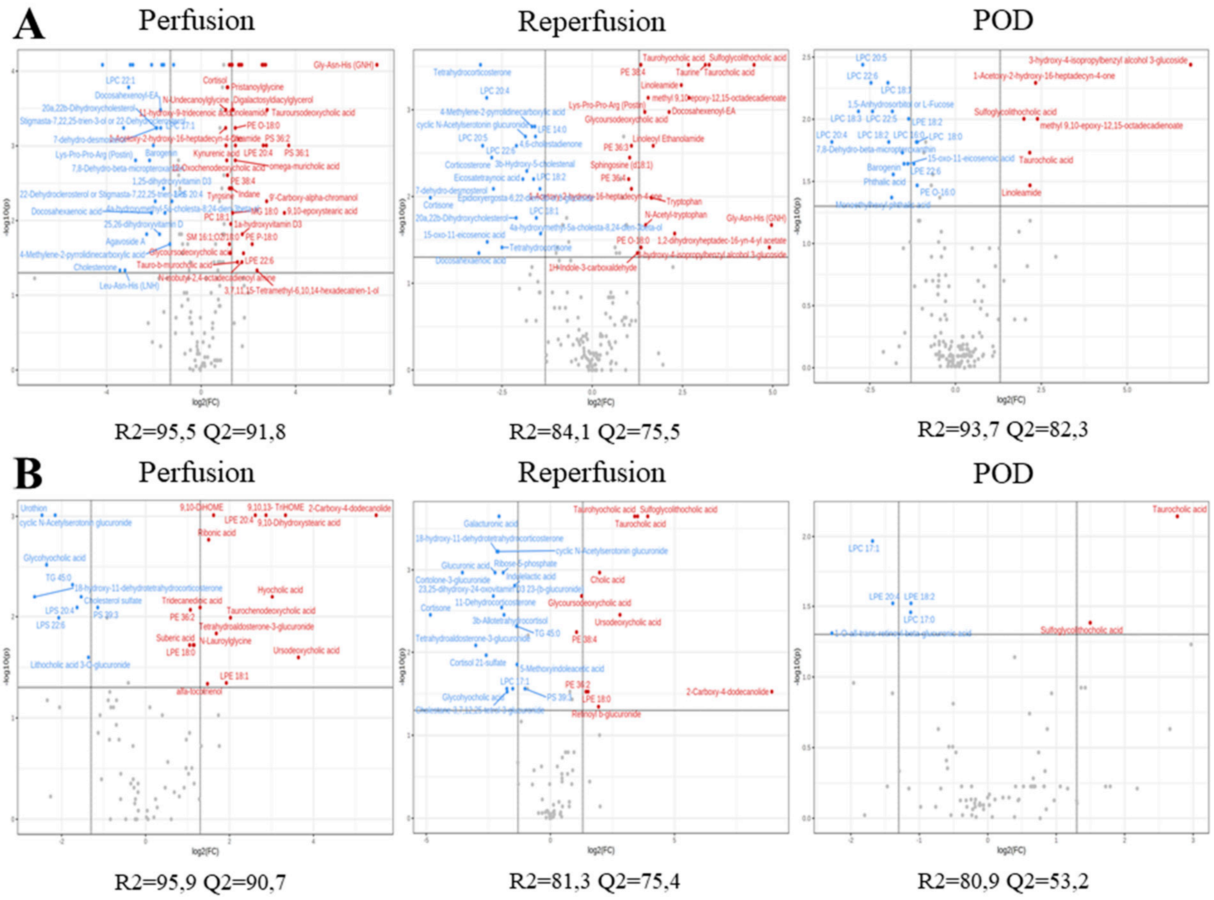

Figure S2. Volcano plots showing the number of metabolites differentiating the 90'DCD group compared to the HBD group in particular time intervals (fold change >2. p< 0.05 with FDR correction) for positive ioniazation mode (A) and negative ionization mode (B). red color - increased level after ischemia. blue color - decreased level after ischemia
